# Avian Resistance to *Campylobacter jejuni* Colonization Is Associated with an Intestinal Immunogene Expression Signature Identified by mRNA Sequencing

**DOI:** 10.1371/journal.pone.0040409

**Published:** 2012-08-01

**Authors:** Sarah Connell, Kieran G. Meade, Brenda Allan, Andrew T. Lloyd, Elaine Kenny, Paul Cormican, Derek W. Morris, Daniel G. Bradley, Cliona O'Farrelly

**Affiliations:** 1 Smurfit Institute of Genetics, University of Dublin, Trinity College, Dublin, Ireland; 2 Teagasc, Animal & Grassland Research and Innovation Centre, Co Meath, Ireland; 3 Vaccine and Infectious Disease Organization, University of Saskatchewan, Saskatoon, Canada; 4 School of Biochemistry and Immunology, University of Dublin, Trinity College, Dublin, Ireland; 5 Trinity Genome Sequencing Laboratory, Institute of Molecular Medicine and Dept. of Psychiatry, University of Dublin, Trinity College, Dublin, Ireland; University of California, Davis, United States of America

## Abstract

*Campylobacter jejuni* is the most common cause of human bacterial gastroenteritis and is associated with several post-infectious manifestations, including onset of the autoimmune neuropathy Guillain-Barré syndrome, causing significant morbidity and mortality. Poorly-cooked chicken meat is the most frequent source of infection as *C. jejuni* colonizes the avian intestine in a commensal relationship. However, not all chickens are equally colonized and resistance seems to be genetically determined. We hypothesize that differences in immune response may contribute to variation in colonization levels between susceptible and resistant birds. Using high-throughput sequencing in an avian infection model, we investigate gene expression associated with resistance or susceptibility to colonization of the gastrointestinal tract with *C. jejuni* and find that gut related immune mechanisms are critical for regulating colonization. Amongst a single population of 300 4-week old chickens, there was clear segregation in levels of *C. jejuni* colonization 48 hours post-exposure. RNAseq analysis of caecal tissue from 14 *C. jejuni*-susceptible and 14 *C. jejuni*-resistant birds generated over 363 million short mRNA sequences which were investigated to identify 219 differentially expressed genes. Significantly higher expression of genes involved in the innate immune response, cytokine signaling, B cell and T cell activation and immunoglobulin production, as well as the renin-angiotensin system was observed in resistant birds, suggesting an early active immune response to *C. jejuni*. Lower expression of these genes in colonized birds suggests suppression or inhibition of a clearing immune response thus facilitating commensal colonization and generating vectors for zoonotic transmission. This study describes biological processes regulating *C. jejuni* colonization of the avian intestine and gives insight into the differential immune mechanisms incited in response to commensal bacteria in general within vertebrate populations. The results reported here illustrate how an exaggerated immune response may be elicited in a subset of the population, which alters host-microbe interactions and inhibits the commensal state, therefore having wider relevance with regard to inflammatory and autoimmune disease.

## Introduction

The healthy vertebrate intestine is densely colonized with a wide range of non-pathogenic microorganisms which include members of all three domains of life – the eukarya, archaea and bacteria. Co-evolution of host and intestinal microbial species over millions of years has promoted beneficial coexistence and interdependency. Local immune homeostasis in the intestine is critical for both host health and commensal survival and at the same time is required to provide effective defense against harmful pathogens. The gastrointestinal immune system is consequently highly specialized and composed of cellular and molecular components with complex functional and regulatory features [1], [2]. Association of the gut microbiota with intestinal autoimmune disease [3]–[5] is therefore not surprising, however recent studies have also implicated the gut commensal microbiota in development of the extraintestinal autoimmune diseases arthritis [6], [7], type I diabetes mellitus [8], [9] and the mouse model of multiple sclerosis [10], [11].

In humans, *C. jejuni* infection elicits an inflammatory response [12]–[17] which regularly causes pathological symptoms. The majority of *C. jejuni* cases are mild, self-limiting and pathology is restricted exclusively to the intestine but the course of infection is not always predictable and it can occasionally spread to other tissues especially in the elderly and immunocompromised, leading to significant morbidity and mortality [18]–[20]. Campylobacteriosis has also been associated with post-infectious sequelae such as Guillain-Barré syndrome [21], [22], its rare variant Miller Fisher Syndrome [23] and reactive arthritis [24].

In contrast to infection in humans, *C. jejuni* does not induce any pathology in chickens and inhabits the lower intestine in a commensal relationship. The principal site of chicken colonization is within the mucus overlying crypts of the caeca, large intestine and cloaca [25]. Histopathological studies reveal no evidence of necrosis and no significant change in crypt architecture [25]–[27]. Although the bacteria are not observed attached to chicken intestinal epithelial cells *in vivo* [25], several *C. jejuni* adhesins have been identified and epithelial cell attachment is believed to be required for successful colonization. Invasion of primary chicken intestinal epithelial cells has been described [28], [29], but *C. jejuni* survives intracellularly for only a short time and subsequently the bacteria evade the cells. Replication is not thought to occur here but most likely happens in chicken intestinal mucus, where it has been demonstrated *in vitro* [29], [30]. Interestingly, due to the contrasting commensal and pathogenic infections which develop in chicken and human, chicken mucin limits attachment and invasion of human primary and cultured intestinal epithelial cells, while in contrast human mucus enhances internalization [28], [31]. Adherence and invasion potential of different *C. jejuni* strains with human Caco-2 cells correlates with colonization ability in the chicken intestine [32], whereas chicken intestinal caecal cell invasion correlates with systemic rather than intestinal colonization level [29]. Even though *C. jejuni* colonizes the avian intestine in an apparently commensal relationship, systemic colonization does occur, with several studies detecting the organism extra-intestinally soon after both intra-cloacal and oral infection in the bursa of Fabricius, spleen and liver/gallbladder [29], [33]–[35]. It has also been found in the thymus, reproductive tract and circulating blood of commercial birds [36]–[38]. Although no pathology is associated with chicken colonization, an intestinal immune response to infection has been illustrated with increased cytokine expression [14], [39]–[42] and toll-like receptor (TLR) activation [43]. Our group has previously carried out global gene expression analysis of the immune response to *C. jejuni* 20 hours post-infection and demonstrated activation of several pathways, including evidence of T cell involvement [44]. Differential caecal expression 7 days after infection of 1-day old chicks has also been investigated both within and between two broiler lines which differ in their susceptibility to colonization [45], [46] and significant induction of the *MAPK* pathway, GTPase-mediated signal transduction and several immune genes, including some indicative of T cell and B cell activity, was found. An increase in circulating macrophages can be observed in peripheral blood 6 hours post-challenge [33]. Heterophil influx into caecal tissues just 1 day post-infection has also been reported [40], although some studies report a lack of heterophil infiltration [29], [33]. These conflicting findings are probably due to differences between chicken lines and age of infection. A systemic immune response to *C. jejuni* involving lymphocyte activation and immunoglobulin production, has been demonstrated in the spleen [47]. Thus an early active immune response seems to be required for colonization and its regulation critical to preventing pathology. Colonization levels within the gut vary substantially between individual chickens and several studies have illustrated that this variation in colonization susceptibility is heritable [48]–[50]. Resistance to caecal colonization was previously shown to be dependent on host cell lineage in three crossbred stocks of commercial chickens [48]. Consistent differential colonization between inbred chicken lines was observed as soon as 24 hours after oral challenge with *C. jejuni*, with results of backcrossing experiments indicating the colonization resistance observed was inherited as an autosomal dominant trait [49]. A paternal genetic effect associated with resistance to colonization has also been reported in a population of commercial broilers [50].

Here, using Illumina high-throughput short-read sequencing of caecal transcriptomes we identify differential expression patterns between mRNA isolated from colonization-resistant and colonization-susceptible birds from a single population of *C. jejuni*-infected chickens. We observe differential expression of genes from several key immune pathways indicating that these immunological mechanisms are fundamental in regulating *C. jejuni* colonization. Significantly increased expression of a range of cytokines and immune effector molecules, T cell and B cell activation and immunoglobulin production are observed. This controlled immune response is accompanied by activation of components of the renin-angiotensin system and appears to confer colonization resistance to a subset of the birds analyzed.

## Results and Discussion

### Infection model separates colonization-resistant and colonization-susceptible birds

**Figure 1:**
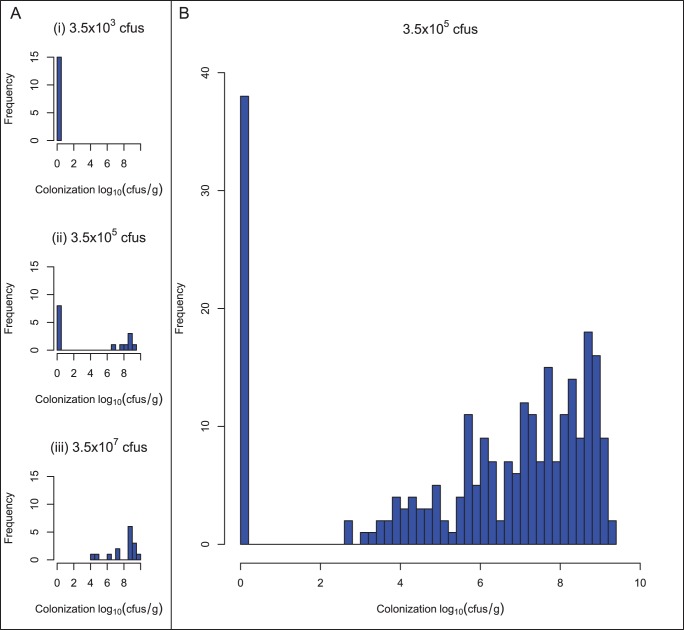
Infection model optimization and distribution of colonization levels. (A) To determine the optimum dose where differential colonization is observed, three groups of fifteen birds were initially inoculated with low (i, 3.5 ×10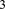), medium (ii, 3.5×10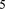) and high (iii, 3.5×10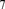) doses of *C. jejuni*, and the colonization status of their caeca estimated after 48 hours. No colonization was detected in any bird after infection with 3.5×10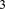 CFU. All birds were colonized after high dosage. Maximum differentiation is achieved after inoculation with the medium dose of 3.5×10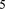 CFU *C. jejuni*. (B) 255 birds were challenged with 3.5×10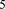 *C. jejuni* and their caecal colonization status determined after 48 hours. No *C. jejuni* colonization could be detected in 38 birds. Colonization levels of the remaining birds varied from 2×10
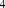
– 4 ×10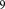 CFU/g with the majority of this group having very high caecal *C. jejuni* levels (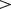
10
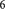
CFU/g *C. jejuni*).

Three groups of 15 chickens were initially inoculated with 3.5×10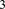 CFU, 3.5×10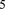 CFU and 3.5×10 CFU *C. jejuni* and their caecal load determined 48 hours after inoculation. No *C. jejuni* growth was detected on plates of caecal content from birds given the lowest dosage of 3.5×10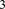 CFU. Growth levels on the plates from medium-dosage birds were highly variable, with 7/15 plates having a very high number of CFU (
6×10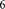 CFU/g) and no growth observed on the remaining 8 plates. All plates from the high-dosage group were colonized with levels varying from 2×10
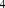
– 4×10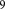 CFU/g. It was therefore determined that the dosage level resulting in optimal differential colonization in the flock was the medium dose of 3.5×10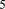 CFU (Figure 1A). After inoculation of the remaining 255 chicks with 3.5×10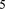 CFU *C. jejuni*, differential colonization was again observed with 38 chicks having no plate colonization and the remaining 217 chicks having colonization levels ranging from 400 CFU/g to 2.5×10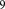 CFU/g (Figure 1B).

### Sequence alignment and transcript level estimation

**Figure 2:**
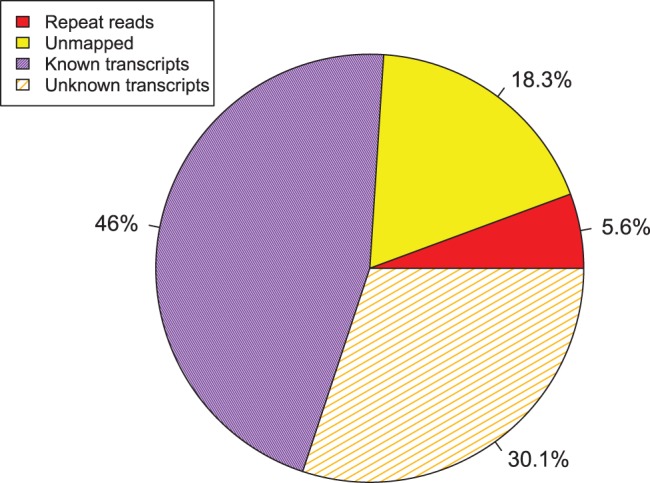
RNAseq read mapping percentage breakdown. 175.31 million (48.2%) of the reads generated from mRNA sequencing could be mapped uniquely to known gene models and therefore could be used in estimation of gene expression levels. 101.28 million reads (27.8%) were mapped successfully to the genome but could not be mapped to NCBI gene models (unknown transcripts, 3.65 Gbp) indicating a necessity for more comprehensive annotation of the chicken transcriptome. Relatively few repeat reads (20.49 million reads, 5.6%) were observed consistent with the low repeat density of the chicken genome [51].

14 nil-colonized and the 14 highest-colonized birds were selected for mRNA sequencing. Two RNAseq libraries were prepared from the first sample, a nil-colonized bird, and each library was sequenced using one lane of an Illumina Genome Analyzer generating an average of 8 million reads per library. Little background noise was observed between the two sequenced lanes and due to the very high correlation and high number of reads obtained, it was determined that one lane was sufficient to sequence each sample for our analysis. The remaining 27 samples were subsequently sequenced allowing one lane per sample. After filtering for low quality reads and primer contamination, 363.75 million 36 bp reads were successfully sequenced and used in the analysis. A high proportion of reads (76.0%) mapped back to the chicken genome and exon-exon boundaries. As expected due to low repeat density within the chicken genome [51], relatively few reads (only 5.6%) mapped to more than one genome region. 27.84% of reads which mapped successfully to the genome did not map to known NCBI gene models (Figure 2). A large dataset of expression level estimates is presented in this study which may facilitate future studies of coexpression dynamics and also substantially facilitate updated annotation of the chicken genome.

### Analysis of gene expression reveals substantial transcriptome differences between susceptible and resistant birds

**Figure 3:**
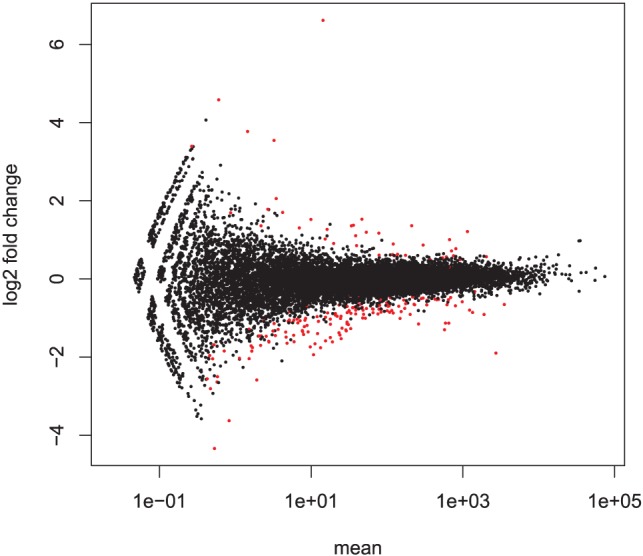
Detection of differentially expressed genes between high-colonized and nil-colonized birds. Log2 fold change is plotted versus mean count numbers reflecting expression level. The R package DESeq was used to compare expression levels between the two groups of high-colonized and nil-colonized chickens and to identify genes displaying significant differential expression using a negative binomial model of the count data. 221 genes exhibiting significant differential expression with p-value 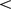
0.01 are highlighted red.

The R package DESeq [52] was used to test for differential expression implementing a negative binomial model of the count data. 41 genes exhibited significant differential expression after adjustment for multiple testing (Benjamini & Hochberg p-value 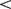
0.01). Functional analysis of these genes did not uncover significant enrichment of any gene ontology (GO) terms or KEGG pathways after adjustment for multiple testing. In order to identify more subtle patterns of differential expression, a relaxed significance threshold of unadjusted p-value 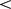 0.01 was implemented and a total of 221 genes exhibited differential expression between high-colonized and nil-colonized birds at this threshold (Figure 3 and Table S1). Two of these genes (*LOC776447* and *LOC431338*) displayed unusually high residual variance quotients compared to genes of similar expression levels. Their measures of significance were therefore considered unreliable and they were excluded from subsequent analysis. The majority (175/219, 80%) of differentially expressed genes exhibited higher expression in nil-colonized relative to high-colonized birds.

**Figure 4:**
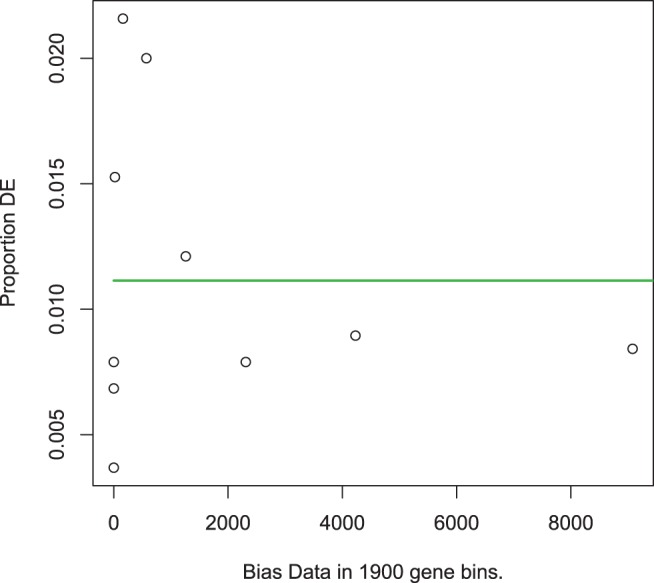
Differential expression as a function of read count. The proportion of genes differentially expressed is plotted against the total number of reads for each gene. This plot investigates whether differential expression is more likely to be detected in genes with a higher number of read counts. The green line is the probability weighting function fitted by Goseq. Flat  =  no bias present and no correction. This illustrates that the method to detect differential expression was robust against bias introduced by differing count numbers.

Analysis of abundance of differentially expressed genes within biological pathways using Goseq [53] did not reveal any bias introduced by differing expression levels (Figure 4), which is in agreement with the even distribution of mean count numbers among significant genes depicted in Figure 3. Functional analysis of these genes using Goseq showed a significant overrepresentation of genes involved in several KEGG pathways and gene ontology (GO) terms (FDR  = 0.05). Differentially expressed genes were also divided into two lists of genes with increased or decreased expression in nil-colonized birds relative to high-colonized birds and analyzed separately. The set of genes with increased expression was also significantly enriched for several GO terms and KEGG pathways but no significant results were obtained with the set of genes with lower expression. Significant results from this analysis are summarized in Table 1. Several immune processes were among the GO categories significantly enriched and indicated an increase in membrane composition and signaling activity in nil-colonized birds, particularly in B cells and T cells. KEGG pathway analysis also revealed differential expression of cytokines, cell adhesion molecules (CAMs) and genes within the renin-angiotensin system (RAS). Although not detected through biological pathway analysis, there was strongly increased expression of immunoglobulin genes in nil-colonized birds (Table 2).

**Table 1:** GO Terms and KEGG pathways significantly enriched in differentially expressed genes.

	+/− in *C.jej*-		+ in *C.jej*−	
GO Term	p-value	FDR	p-value	FDR
Membrane	8.3e−07	0.004	2.8e−05	0.023
External side of plasma membrane	5.4e−06	0.013	1.5e−06	0.007
B cell receptor signaling pathway.	1.4e−05	0.023	7.2e−06	0.018
Positive regulation of interleukin-2 biosynthetic process	2.8e−05	0.034	1.4e−05	0.023
Positive regulation of calcium-mediated signaling	4.8e−05	0.048	2.5e−05	0.023
Cell surface receptor linked signaling pathway	6.7e−05	0.054	2.8e−05	0.023
Positive regulation of peptidyl-tyrosine phosphorylation	7.6e−05	0.054	3.9e−05	0.026
T cell receptor complex	1.3e−04	0.063	8.1e−05	0.040
Transmembrane receptor activity	9.9e−05	0.061	4.1e−05	0.026
B cell receptor complex	1.3e−04	0.063	8.1e−05	0.040

KEGG pathway	p-value	FDR	p-value	FDR
Renin-angiotensin system	1.0e−05	0.002	4.3e−06	0.001
Cytokine-cytokine receptor interaction	1.6e−04	0.012	1.2e−03	0.073
Cell adhesion molecules	4.9e−04	0.024	1.5e−03	0.073

All p-values and FDRs are shown for terms and pathways exhibiting significant enrichment in all significant genes or in the set of significant genes with increased expression in nil-colonized birds relative to high-colonized birds. Results clearly demonstrate a significantly higher expression of genes affecting cell membrane composition and several immune pathways in nil-colonized birds relative to high-colonized birds.

**Table 2:** Immunoglobulins and non-categorized immune genes exhibiting differential expression between resistant and susceptible birds.

Gene ID	Name	Descriptor	Fold	+ 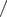 −	p-value
Immunoglobulins					
427703	*LOC427703*	Immunoglobulin, light chain, lambda, psi-V4	3.8	+	4.6e−06
769305	*LOC769305*	Ig lambda chain V-1 region-like	3.7	+	6.2e−06
772169	*LOC772169*	Similar to Ig alpha chain	3.7	+	1.3e−03
769305	*LOC769305*	Ig lambda chain V-1 region-like	3.7	+	6.2e−06
430014	*LOC430014*	Ig heavy chain V-III region VH26-like	3.6	+	1.0e−04
769327	*LOC769327*	Immunoglobulin, light chain, lambda, psi-V12	3.4	+	2.2e−06
416929	*LOC416929*	Immunoglobulin, light chain, lambda, psi-V7	3.3	+	3.5e−07
769283	*LOC769283*	Immunoglobulin, light chain, lambda, psi-V8	3.3	+	4.3e−06
430015	*LOC430015*	Ig heavy chain V-III region VH26-like	3.0	+	8.2e−03
X07174.1	X07174.1	mRNA for IgY H-chain	3.0	+	2.4e−19
430017	*LOC430017*	Ig heavy chain V-III region VH26-like	2.9	+	5.6e−07
431191	*VH1*	Immunoglobulin heavy chain, variable	2.9	+	2.5e−04
374117	*IGJ*	Immunoglobulin J, linker protein for Ig alpha and mu	2.8	+	1.5e−17
416930	*LOC416930*	Ig lambda chain V-1 region-like	2.7	+	9.0e−06
431524	*LOC431524*	Ig heavy chain V-III region VH26-like	2.6	+	2.8e−04
425694	*LOC425694*	Ig heavy chain V-III region VH26-like	2.3	+	5.7e−03
416928	*IGLL1*	IGLV immunoglobulin lambda-like polypeptide 1	2.1	+	4.8e−09
S40610.1	S40610.1	mRNA for IgA H = Ig alpha heavy chain	2.0	+	7.4e−08
X01613.1	X01613.1	mRNA for mu Ig heavy chain C region	1.9	+	4.1e−07
Immune Genes					
426862	*IVNS1ABP*	influenza virus NS1A binding protein	4.3	+	2.6e−03
430678	*CHIR-B5*	Immunoglobulin-like receptor CHIR-B5	4.0	+	1.5e−03
374055	*TRAF5*	TNF receptor-associated factor 5	2.1	+	2.8e−04
426274	*TLR1LA*	Toll-like receptor 1LA	2.1	+	2.5e−03
395669	*PLEK*	Pleckstrin	2.0	+	3.1e−03
426106	*LOC426106*	C-type lectin domain family 17 member A-like	1.9	+	4.1e−04
396218	*LYZ*	Lysozyme	1.9	+	2.5e−04
396495	*CXCLi2*	Inflammatory chemokine (previously IL8)	1.9	−	6.9e−03
424121	*ITGA4*	Integrin, alpha 4 (antigen CD49D)	1.8	+	3.6e−03
423790	*IFIT5*	Interferon-induced protein with TPRs 5	1.8	−	1.6e−04
419862	*TRAF3IP3*	TRAF3 interacting protein 3	1.5	+	1.4e−03
395313	*MX1*	Myxovirus (influenza virus) resistance 1	1.5	−	4.3e−03
416546	*NOXO1*	NADPH oxidase organizer 1	1.4	−	2.4e−03

Genes are ordered according to fold-difference and the direction of fold-difference is shown to indicate either increased (+) or decreased (−) expression in resistant relative to susceptible birds. The unique NCBI Gene ID for each gene is shown in the first column. All of the immunoglobulin genes, including the immunoglobulin lambda chain gene *IGLL1*, all four annotated immunoglobulin V-type pseudogenes and the immunoglobulin J gene (*IGJ*) have significantly greater expression in resistant birds. Additionally the IgM (X01613.1), IgA (S40610.1) and IgY (X07174.1) heavy chain transcripts have significantly higher expression, indicating increased IgM, IgA and IgY production. Differentially expressed genes with immunological functions which are not directly associated with lymphocyte activation or immunoglobulin production are also shown here. Innate immune genes toll-like receptor 1LA (*TLR1LA*) and lysozyme (*LYZ*) display increased expression in resistant birds whereas the proinflammatory chemokine *CXCLi2* and the interferon-stimulated gene myxovirus resistance 1 (*MX1*) demonstrate lower expression in resistant birds.

Another notable aspect of the increased immune response observed in nil-colonized birds was significantly greater expression of innate immune genes encoding antibacterial molecules such as lysozyme (*LYZ*) which degrades bacterial cell walls, NADPH oxidase organizer 1 (*NOXO1*) which regulates respiratory burst and toll-like receptor 1LA (*TLR1LA*) (Table 2). However, the proinflammatory chemokine *CXCLi2* (previously *IL8*) displays significantly lower expression in nil-colonized relative to high-colonized birds. Numerous studies have demonstrated *CXCLi2/IL8* induction in response to *C. jejuni* in both human [12], [13], [15], [16], [42], [54]–[57] and chicken [14], [39]–[41]. We believe the lower expression of *CXCLi2* in nil-colonized birds here is due to clearance of the bacteria through the different innate immune responses described and a consequently lower inflammatory response to *C. jejuni* exposure. Myxovirus (influenza virus) resistance 1 (*MX1*), which limits replication of influenza and other viruses dependent on allele-specific expression [58]–[61] also displays significantly lower expression in resistant birds. *MX1* is an interferon-stimulated gene (ISG) and its expression is an indicator of IFN-a/b activity. Type I IFN responses are typically associated with viral infections, but several recent reports demonstrate their induction in reponse to bacterial infection, with the effect of either facilitating or inhibiting infection [62]–[66]. We believe the significantly higher expression of this gene along with higher expression of Interferon-induced protein with TPRs (*IFIT5*) and lower expression of Interferon Regulatory Factor 4 (*IRF4*) in colonized birds suggests a type I IFN response in these birds which must cope with intracellular invasion. Increased expression of *IFN-b* and *MX1* was also observed in colonized birds in a study of caecal expression 7 days after infection of a population of 1-day old chicks [46] and IFN-b secretion has been described in human dendritic cells in response to *C. jejuni* [67].

### Immunoglobulin production

Many genes involved in production of immunoglobulin, which limits penetration of the epithelial cell layer by commensal bacteria, were found to have increased expression in nil-colonized birds (Table 2). The main immunoglobulin lambda chain gene (*IGLL1*) and a similar lambda chain gene (*LOC769305*) are significantly increased 2.1-fold and 3.7-fold respectively, as are all four upstream immunoglobulin V-type pseudogenes which are annotated on the reference genome, their fold-changes ranging from 3.3-fold to 3.8-fold. Several genes sharing homology with heavy chain variable regions are also significantly increased. In addition, the gene encoding IgJ linker protein which is required for IgA and IgM polymerization is increased 2.8-fold. The genes encoding the immunoglobulin heavy chains are not annotated in the chicken genome. In order to estimate their expression levels, alignment was conducted to the mRNA sequences of the heavy chains of IgM (X0613.1), IgA (S40610.1) and IgY (X07174.1). Expression of IgM, IgA and IgY heavy chains was significantly higher (1.9-fold, 2.0-fold and 3.0-fold respectively) in resistant birds (Table 2 and Table S1). Together, these results indicate that there is increased production of dimeric secretory IgA, IgM and IgY in the group of nil-colonized birds which is associated with resistance to colonization.

B cells in the gastrointestinal mucosa differentiate into plasma cells which secrete large amounts of immunoglobulin to both protect against infiltration of pathogenic bacteria and to control rapidly replicating commensal colonization. T cell-dependent B cell activation and high-affinity immunoglobulin production typically takes 3–5 days, which may be too slow to effectively protect the intestine from constant immune challenge. Extrafollicular B1 cells from the peritoneal cavity and intestinal lamina propria which secrete polyreactive low-affinity immunoglobulin can respond more rapidly with T cell-independent production of IgM and class-switched IgA and IgG [68]–[71]. Although the presence of B1 cells in the chicken is not certain, all avian B cells express *CD5*, similar to B1 cells, and it has been proposed that avian B cells have some functional and developmental similarity to mammalian B1 cells [72], [73]. Production of specific immunoglobulin after *C. jejuni* colonization of the chicken caecum has previously been shown many weeks post-infection [74], [75] but the results reported here suggest that a more rapid T cell-independent pathway of immunoglobulin production may be induced in response to infection. We believe the increased expression of IgM, IgA and IgG observed is due to increased migration, proliferation and activity of B cells in response to infection in a manner similar to that seen in mammalian B1 cells. Although it is possible this differential expression may represent a dampening down of B cell activity in colonized birds corresponding to induction of the commensal state, we believe this is very unlikely as the intestine responds to commensal colonization with increased immunoglobulin production [68], [76].

The increased immunoglobulin production observed in nil-colonized birds here affirms that an immune response has been activated in these birds which ultimately confers resistance to *C. jejuni* colonization. Nil-colonized birds will therefore be referred to as resistant from this point in our discussion.

### Lymphocyte differentiation and activation

In accordance with the increase in immunoglobulin production observed in resistant birds, genes involved in B cell receptor (BCR) signaling also displayed significantly increased expression. BCR-mediated signaling is crucial for multiple stages of B cell development, B cell selection and activation in response to antigen and initiation of immunoglobulin diversification through somatic hypermutation [77], [78]. Several genes critical for B cell development and activation displayed increased expression in resistant birds e.g. *PAX5*, *IRF4*, *AID*, *CD45*, *CD79B*, *DOCK8*, *POU2AF1*, *VPREB3* and the two avian *CD72* paralogues (Table 3). This development and activation of B cells is characteristic of the immune response to bacteria, including commensal bacteria. Additionally, the expression of all three *CXCL13* genes and the receptor *CXCR5*, whose products interact to control the migration of B cells are all significantly increased in resistant birds also indicating recruitment of B cells in response to the bacterium.

**Table 3:** Genes involved in lymphocyte development and function exhibiting significant difference in expression between resistant and susceptible birds.

Gene ID	Name	Descriptor	Fold	+ 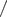 −	p-value
Lymphocytes					
395090	*TNFRSF8*	TNF receptor superfamily, member 8	3.2	+	1.1e−06
418426	*BTLA*	B and T lymphocyte associated	2.6	+	3.7e−06
419854	*CR2*	Complement component (3d 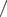 Epstein Barr virus) rec 2	2.1	+	4.5e−07
416586	*IL21R*	Interleukin 21 receptor	2.0	+	2.4e−04
427612	*DOCK2*	Dedicator of cytokinesis 2	1.9	+	6.6e−04
374270	*IL16*	IL16 (lymphocyte chemoattractant factor)	1.9	+	1.3e−03
428315	*CCR7*	Chemokine (C-C motif) receptor 7	1.7	+	2.6e−03
422827	*CD38*	CD38 molecule	1.5	+	5.3e−03
B Cells					
422511	*LOC422511*	CXCL13L3/CXCL13c	3.2	+	1.2e−04
768355	*LOC768355*	B-cell differentiation antigen CD72-like	2.9	+	3.2e−03
418257	*CD72*	CD72 molecule (2)	2.3	+	2.9e−03
427415	*PAX5*	Paired box 5	2.2	+	1.3e−06
387330	*B6.1*	B cell marker chB6	2.1	+	3.6e−07
396098	*IRF4*	Interferon regulatory factor 4	2.0	+	3.0e−05
374179	*CIITA*	Class II, MHC, transactivator	2.0	+	2.5e−03
427676	*POU2AF1*	POU class 2 associating factor 1	1.9	+	4.0e−06
373994	*CXCR5*	Chemokine (C-X-C motif) receptor 5	1.9	+	2.3e−05
419784	*VPREB3*	Pre-B lymphocyte gene 3	1.9	+	2.3e−04
416932	*CD72*	CD72 molecule (1)	1.8	+	1.1e−04
395923	*DOCK8*	Dedicator of cytokinesis 8	1.8	+	2.9e−04
427348	*PTPRC*	CD45 – protein tyrosine phosphatase	1.6	+	1.5e−03
386580	*LOC422509*	CXCL13L1/CXCL13a	1.5	+	6.2e−03
422509	*LOC422510*	CXCL13L2/CXCL13b	1.5	+	9.7e−03
422510	*CD79B*	CD79b molecule, immunoglobulin-associated beta	1.4	+	8.0e−04
419940	*AICDA*	Activation-induced cytidine deaminase	1.3	+	1.0e−03
T Cells					
769256	*IL17REL*	Interleukin 17 receptor E-like	3.2	+	4.4e−04
769224	*THEMIS*	Thymocyte selection associated	2.8	+	4.1e−04
418412	*TRAT1*	T cell receptor associated transmembrane adaptor 1	2.3	+	7.7e−03
424106	*CTLA4*	Cytotoxic T-lymphocyte-associated protein 4	2.2	+	3.8e−03
769232	*LOC769232*	Similar to T cell receptor alpha	2.1	+	1.2e−05
769716	*LOC769716*	Class I histocompatibility antigen	2.1	+	6.2e−04
416247	*ITK*	IL2-inducible T-cell kinase	2.0	+	5.0e−03
418535	*UBASH3A*	Ubiquitin associated and SH3 domain containing, A	1.9	+	5.4e−04
424187	*DPP4*	Dipeptidyl-peptidase 4 (CD26)	1.8	+	5.9e−05
395362	*CD4*	CD4 molecule	1.8	+	2.8e−03
396062	*CD3E*	CD3e molecule, epsilon (CD3-TCR complex)	1.5	+	5.6e−03
417058	*LOC417058*	Class I histocompatibility antigen	1.5	−	5.8e−03
396518	*CD3D*	CD3d molecule, delta (CD3-TCR complex)	1.4	+	5.9e−03

Genes which are involved in lymphocyte differentiation, migration and activation are separated into those which are more closely associated with B cells and T cells. Genes are ordered according to fold-difference and specified as displaying either increased (+) or decreased (−) expression in resistant relative to susceptible birds. There is clear evidence for increased B cell and T cell development and activation in resistant birds, with most genes demonstrating greater expression (+).

We observe substantial evidence for T cell proliferation and activation in resistant birds including expression of *CD4*, *CTLA4*, *CD3D*, *CD3E* and T cell receptor alpha genes (Table 3), which are consistent with previous findings of possible T cell activation using microarrays and RT-PCR [44], [45]. Typically, the immune response to commensal bacteria within the mucosa involves T cell-independent production of immunoglobulin in extrafollicular B cells in order to limit penetration of the bacteria [79]. Although T cell-dependent IgA production has also been described [80], these mechanisms are unlikely to be involved two days post-exposure. Due to the significant differential expression of a T cell receptor alpha gene observed, this T cell response is likely to reflect greater activity of 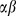 T cells. However, a T cell receptor gamma gene did also exhibit greater expression in resistant birds which almost reached significance (*LOC776306*, 1.65-fold, p-value  = 0.032) so 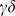 involvement can not be completely excluded. Similarly, although only the *CD4* gene displayed significant differential expression, the *CD8A* and *CD8B* genes demonstrated increased expression at levels almost reaching significance (Table S1). The T cell response observed may reflect greater activity of regulatory or helper T cells, both of which play a crucial role in intestinal homeostasis. Notably, the IL17 receptor E-like gene is increased over 3-fold, perhaps indicating increased response potential to T
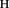
17 cells.

Several important indicators of lymphocyte activity common to both B cells and T cells, such as *TNFRSF8*, *CCR7*, *IL21R* and *CD38* also display significantly greater expression in resistant birds (Table 3). In addition, significant evidence for positive regulation of IL2 biosynthesis is seen. IL2 is fundamental for regulatory T cell development, cytotoxic T cell differentiation, development of immunological memory, discrimination between self and non-self and can promote B cell proliferation and production of immunoglobulin [81], [82]. Its production is consistent with the other aspects of an increased immune response seen in resistant birds.

Substantial differences in expression of genes encoding cell membrane components and cell adhesion molecules are seen between resistant and susceptible birds. These differences are most probably due to the change in cellular composition of the tissue and a greater density of lymphocytes, although this may also be reflective of greater cell-cell contact and creation of a stronger membrane barrier against bacterial infiltration.

### Renin-angiotensin system activation

Increased expression of genes involved in the renin-angiotensin system is seen in resistant relative to susceptible birds. This pathway principally regulates blood pressure and renal function. However, numerous studies have highlighted involvement of members of the renin-angiotensin system in inflammatory processes including regulation of T cell function [83], recruitment of inflammatory cells [84], production of reactive oxygen species [85], and autoimmune inflammation of the central nervous system [86], [87]. One of the central enzymes, Angiotensin-Converting Enzyme (ACE), has been shown to regulate DC maturation and TH1 cell development in response to Trypanosome infection in the mouse [88]. *ACE* overexpression in myelomonocytic lineage cells also confers an increased proinflammatory phenotype in mice which is hyperresponsive to challenge with models of lymphoma and melanoma [89], [90]. In addition, *ACE* overexpression increased the immune response to bacterial infection with *Listeria monocytogenes* and methicillin-resistant *Staphylococcus aureus* (MRSA) *in vitro* and *in vivo* [91]. Studies in the animal model of MS, experimental autoimmune encephalomyelitis (EAE), suggest ACE induces autoreactive T
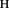
1 and T
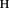
17 cells and suppresses regulatory T cells [86], [87] thus playing a significant role in autoimmune inflammation. Higher expression of the gene encoding this enzyme and other crucial genes within the renin-angiotensin system is observed here in resistant relative to susceptible birds (Table 4). This is in keeping with the other findings of an exaggerated immune response in resistant birds which obviates establishment of bacterial colonization.

**Table 4:** Genes involved in the renin-angiotensin system (RAS) exhibiting differential expression between resistant and susceptible birds.

Gene ID	Name	Descriptor	Fold	+ 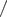 −	p-value
Renin-angiotensin System					
428771	*ENPEP*	Glutamyl aminopeptidase (aminopeptidase A)	2.0	+	2.3e−07
418623	*ACE2*	Angiotensin I converting enzyme 2	1.4	+	4.6e−04
425031	*MME*	Membrane metallo-endopeptidase	1.2	+	4.5e−03
419953	*ACE1*	Angiotensin I converting enzyme 1	1.2	+	5.1e−03

Genes are again specified as displaying either increased (+) or decreased (−) expression in resistant relative to susceptible birds. *ACE1*, *ACE2*, *ENPEP* and *MME* are crucial components of the renin-angiotensin system and all display significantly higher expression in resistant relative to susceptible birds, indicating increased activity of this pathway.

### Summary

In this study, we have demonstrated differential expression of genes involved in the innate immune response and lymphocyte activation associated with resistance to *C. jejuni* colonization in the chicken caecum. We believe the increased expression of genes involved in immunoglobulin production and B cell activity in resistant birds is due to migration, proliferation and activity of B1-like cells in response to *C. jejuni*. B1 cells can respond to immune challenge with a more rapid immunoglobulin response than the classical pathway involving somatic hypermutation and antigen-specific clonal expansion mediated by T cells. This B1 cell alternative activation pathway occurs in the absence of T cell help (although some T cells may play a role in its regulation) in response to T cell-independent antigens such as lipopolysaccharide, hypomethylated CpG-rich DNA, and other conserved microbial signatures which are recognized by TLRs. Strong evidence of increased T cell proliferation and activation is also seen in resistant birds, which is in agreement with previous reports of T cell activity subsequent to *C. jejuni* infection. This is most likely indicative of involvement of regulatory or helper T cells in the response to infection as both contribute significantly to the maintenance of intestinal homeostasis. In addition, significantly higher expression of *ACE* and other genes within the renin-angiotensin system is demonstrated in resistant birds. We believe this pathway is involved in amplifying the innate immune response and may be induced in response to bacterial infection, although it is possible this differential expression was present prior to infection. Further study of this pathway's involvement may be of particular relevance due to association of human *C. jejuni* infection with postinfectious onset of several autoimmune diseases.

## Materials and Methods

### Ethics Statement

This research was performed in strict accordance with the standards set by the Canadian Council on Animal Care. The protocol was approved by the Ethics Committee of the Vaccine And Infectious Disease Organization, University of Saskatchewan. Every effort was made to minimize suffering.

### Infection model, microbiology and sample collection

*C. jejuni* strain NCTC11168v1 was grown on Meuller Hinton (MH) agar plates. The cells were resuspended in saline and viable bacterial numbers were determined on MH agar plates. 300 Barred Rock chickens were obtained from an unselected university flock, maintained by the University of Saskatchewan, Canada at one day of age. The hatchery boxes were swabbed and cultured for *C. jejuni* (on Karmali agar, under mixed gas, conditions at 43°C for 48 hours). The birds were reared in an animal pathogen Containment Level 2 isolation facility at the Vaccine and Infectious Disease Organization (VIDO). At 14 and 21 days of age, 20 birds were randomly chosen and cloacal swabs taken and cultured to ensure they were *C. jejuni*-free. On day 28 all birds were sampled by cloacal swabs and cultured. All birds were culture negative. As chickens were inoculated at 4 weeks of age, the immune system should be fully mature [92]–[95] and any possible anti-*C. jejuni* maternal antibodies not present [96]. In phase I of the infection experiment, 45 birds were separated into three groups of fifteen and challenged orally with suspensions of 3.5×10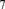 CFU, 3.5×10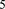 CFU and 3.5×10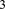 CFU *C. jejuni* estimated by OD600 measurement. The level of *C. jejuni* colonization of the caecum was determined 48 hours post-inoculation. Each bird was euthanized, caecal contents removed, diluted to 0.1 g/ml and five serial 10-fold dilutions created. 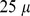
l of each dilution was spread on agar plates and incubated at 42°C. After 48 hours incubation the number of colony-forming units were determined for each dilution. The limit of detection for this analysis was 400 CFU/g. Subsequent to CFU quantification, phase II of the infection experiment was conducted and the remaining 255 birds were inoculated with 3.5×10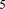 CFU *C. jejuni* as this was the dose where optimum colonization differentiation was achieved. Caecal content samples were again taken for microbiological culture on selectable media for quantitative analysis. Caecal tissue samples were immediately snap-frozen and stored at −80°C until mRNA isolation.

### mRNA sequencing

Following quantitative analysis of colonization levels, whole caecum samples from fourteen nil-colonized and the fourteen highest-colonized birds were selected for mRNA sequencing. Total RNA was initially extracted from each sample with Trizol using the Qiagen RNeasy Lipid purification kit and diluted to 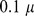
g/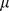
l. All instructions in the Illumina RNA-seq protocol (Part # 1004898 Rev. A September 2008) were followed to purify mRNA and prepare samples for Illumina high-throughput sequencing. Poly-A mRNA was first isolated from total RNA using oligodT beads. The mRNA was then fragmented using divalent cations at 94°C. mRNA was converted to cDNA, blunt-ended at both ends using T4 DNA polymerase and Klenow DNA polymerase. A single A base was then added to the 3′ end to prepare for ligation to adapter sequence. cDNA templates were then size-selected by visualization and excision from agarose gel and purified. The resulting cDNA fragments were PCR-amplified and sequenced generating 40-bp reads using the Illumina Genome Analyzer.

### Transcriptome alignment

Sequence image files were converted to sequence using Illumina Pipeline v1.4. Sequence reads were initially filtered for low-quality sequence and primer contamination. Each read was also trimmed by 4 bp at the 3′ end to eliminate sequences of poor quality towards the ends of the reads. To map sequences back to the transcriptome a collection of sequences representing potential splice crossing reads was created from the reference chicken genome v2.1 [51] with exon coordinates from all NCBI known gene models using Erange v3.3 [97]. The mRNA sequences encoding the heavy chains of IgM (X0613.1), IgA (S40610.1) and IgY (X07174.1) were also included. All filtered 36-bp reads were aligned with Bowtie v0.10.1 [98] to the expanded genome comprising the chicken genome, the collection of all exon-exon fasta sequences and the heavy chain mRNA sequences allowing for 3 mismatches per read and excluding reads that map to more than one position. For each individual lane, the number of counts falling on each gene model was determined with Erange using NCBI known gene models. Two lanes of the Genome Analyzer were used to sequence mRNA from the first bird analyzed – a nil-colonized chicken. The magnitude of experimental background noise between these two technical replicates was investigated by comparison to the hypergeometric distribution, as described in Marioni et al [99], and high correlation between samples was observed accompanied by a high number of reads mapping to known gene models. Therefore one lane of the Genome Analyzer was subsequently used to sequence selected polyA-mRNA from each of the 27 remaining individuals.

### Identification and functional analysis of differentially expressed genes

To compare expression counts between the two groups of high-colonized and nil-colonized samples, a method for testing of differential expression where the count data are modeled with negative binomial distributions was employed in the bioconductor package DEseq v1.0.6 [52] within the R statistical environment. The cut-off used to determine significant differential expression was p-value 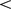
0.01. Fold-changes were estimated by first normalizing each read count per gene by the total number of reads per lane to obtain a RPKM measure. Functional analysis to detect enrichment of GO terms and KEGG pathways within the set of significantly differentially expressed genes was conducted using Goseq v1.0.3 [53]. To account for any selection bias of pathways arising from overrepresentation of long or overly-expressed transcripts, the dataset was corrected for read count bias.

## Supporting Information

Table S1 **Analysis of differential gene expression.** Genes are identified by their unique Entrez GeneID. The CGNC gene symbol and short descriptor are also provided. Average normalized gene counts for low-colonized and high-colonized birds and the fold-change between these two groups are shown. The direction of fold-change is indicated: ‘+’ reflects higher expression and ‘−’ reflects lower expression in low-colonized relative to high-colonized birds. The p-values and adjusted p-values (Benjamini & Hochberg) for each gene are shown. Although GeneID 776447 and GeneID 431338 exhibit significant differential expression, they display high residual variance and their significance estimates are therefore unreliable.
(XLSX)
